# Community activities and their association with social isolation in rural Japan

**DOI:** 10.3389/fpubh.2025.1697377

**Published:** 2026-01-05

**Authors:** Sae Nakaoka, Hiromi Kawasaki, Satoko Yamasaki, Bethel Fekede Menuta, Misaki Shiraishi, Yuan Li

**Affiliations:** Graduate School of Biomedical and Health Sciences, Hiroshima University, Hiroshima, Japan

**Keywords:** community activity, cross-sectional study, isolation, loneliness, rural area

## Abstract

**Background:**

Isolation and subjective feelings of loneliness pose a threat to health, and measures have been taken to address both. However, the decline of communities because of social change has accelerated, and daily support for isolation is lacking. The support offered is not sufficiently effective for isolated individuals, and improvements are needed. This study aimed to identify the characteristics of isolated individuals.

**Methods:**

Survey forms were sent to 1,500 residents aged ≥20 years residing in Sera Town, Hiroshima Prefecture, Japan. This study used secondary data from the communication questionnaire responses. The survey items included basic attributes, as well as measures of isolation, subjective feelings of loneliness, and community activities. Isolation was classified using a scale, and associations with participant characteristics were analyzed using the χ^2^ tests, Kruskal–Wallis tests and Mann–Whitney U tests. A logistic regression analysis was performed to determine the comprehensive relationships between isolation and states. Finally, community activity status was examined specifically among isolated individuals.

**Results:**

The analysis included 689 residents who responded to the survey completely. Of these, 203 were classified as isolated and 486 as non-isolated. The analysis revealed significant associations between isolation and health status, life satisfaction, frequency of working, subjective feelings of loneliness, and community activities. In terms of comprehensive relationships, isolation was associated with fewer frequent outings, higher level of subjective feelings of loneliness, and lower participation in community activities. Isolated individuals considered activities such as helping others, other volunteers, and hobbies to be meaningful and expressed a strong desire to participate in them.

**Conclusion:**

Isolation suggested to be to associated with frequent outings, subjective feelings of loneliness, and participation in community activities. Isolated individuals had less frequent outings, a high level of subjective feelings of loneliness, and did not participate in community activities. Community activities in which isolated individuals want to participate were suggested to have clear goals such as helping others and hobbies. Our findings suggest to be a promising targets for future interventional and evaluation studies.

## 1 Introduction

Isolation and subjective feelings of loneliness are associated with poor mental health, low self-efficacy, and self-esteem (1). Both of these negatively impact physical health and are associated with an equivalent mortality risk than lifestyle-related factors, such as smoking, alcohol consumption, and obesity (2). The Japanese government enacted the Act on the Promotion of Policy for Loneliness and Isolation and formulated a plan to address this issue (3). The government of Japan has recognized isolation and subjective feelings of loneliness as serious and urgent issues, exacerbated by social changes that have led to a lack of interpersonal and societal engagement. Social capital consists of trust, norms, and networks (4). The theory is categorized into bonding, bridging, and linking social capital, each playing a distinct role in reducing psychological distress (5). Traditionally, Japan had a strong sense of community camaraderie, with family structure dominated by three generations—grandparents, parents, and children—living together. Such three-generation families existed throughout the region. From the late 1960s, Japanese industrial structures changed, leading to population overflow in urban areas. Changing lifestyles have resulted in an increase in the number of single-person households (6). Additionally, the increasing age at first marriage and the increase in the number of unmarried individuals have resulted in a declining birthrate and an aging population (6). Traditional Japanese residential communities have weakened bonds and have declined. The decline of existing communities has led to problems of isolation and subjective feelings of loneliness. Due to address the decline of existing communities, the government developed a system in collaboration with administrations and clients, increased the number of consultation methods for isolated individuals and families and encouraged cooperation with existing organizations (7). The Japanese government established a Community-based Integrated Care System (6) and has promoted self-help and mutual aid (8). It implemented a system linking individuals with administrative bodies, enabling them to utilize welfare programs and systems when required. In communities where individuals are in need, neighbors supporting one another fostered a sense of mutual aid among residents (9). However, the decline of communities in recent years has made it difficult to increase the awareness regarding mutual aid for people experiencing subjective feelings of loneliness or isolation. Individuals who feel lonely or are isolated require ongoing and intentional support. Participating in shared activities helps create a connection. Connections between individuals help to widen their social networks and link many social resources. Examples of support for isolation and subjective feelings of loneliness in different countries include self-help cafés, children’s cafeterias and community spaces attached to commercial facilities offering health counseling in Japan (10), and DIY activities for retired men in Australia (11), and cafés in the UK (12) where people can talk casually with others. These initiatives represent measures to prevent isolation and subjective feelings of loneliness by creating places where people can gather. Isolation and subjective feelings of loneliness have long been regarded as the same issue. Various studies have examined this issue, and certain solutions have been considered. However, isolation and subjective feelings of loneliness are two different concepts. Isolation is defined as minimal or no contact with family and community (13), a visible lack of strong and cooperative social networks (14), and insufficient quality and quantity of social relationships with other people at the individual, group, and community levels (15). Feelings of loneliness is a subjective emotion defined as an unwanted feeling due to a lack or loss of companionship (13) or a perceived absence of meaningful social connections (14). Supporters cannot directly perceive whether a recipient is lonely, but isolation can be objectively assessed; thus, supporters can carefully observe signs of isolation in those they assist and gather information. Isolation can contribute to serious social issues, including suicide and abuse; therefore, improvement and preventive interventions are essential. Various factors, including the individual’s life background and social circumstances, can contribute to isolation, and the most effective initial approach to addressing isolation remains unclear. This study aimed to identify the characteristics of isolated individuals.

## 2 Materials and methods

### 2.1 Design and sample

This study is a cross-sectional study. The sample size calculation was performed using G*Power 3.1.9.7 (16, 17). Assuming a two-tailed lognormal test with an effect size of 1.2, a significance level of 5%, and a power of 80%, the required sample size was calculated to be 501 cases.

### 2.2 Target population

This survey was designed to assess mutual aid measures in Sera Town, Hiroshima Prefecture, Japan, and the results were used as secondary data for this study.

Using two-stage stratified random sampling by age group and residential district, the town hall selected 1,500 residents, due to unknown addresses forms were sent to 1,490 individuals, of whom 708 responded to the survey (Table 1). The survey response rate was 47.5%. Of these, 689 people (334 males and 355 females) who completed the questionnaire were included in the analysis. The gender and age distribution of subjects for analysis were presented in Table 2.

**Table 1:** Respondents who received the survey.

Age	Male		Female		Unknown
	Survey participants	Respondents of Survey	Survey participants	Respondents of Survey	
20s	57	6 (10.5%)	58	25 (43.1%)	
30s	67	19 (28.4%)	67	28 (41.8%)	
40s	95	31 (32.6%)	94	39 (41.5%)	
50s	99	34 (34.3%)	99	42 (42.4%)	
60s	130	64 (49.2%)	131	70 (53.4%)	
70s	157	98 (62.4%)	156	106 (67.9%)	
>80s	145	89 (61.4%)	145	55 (37.9%)	
Total	750	341 (45.5%)	750	365 (48.7%)	2

**Table 2:** The gender and age distribution of subjects for analysis.

Age	Total	Male	Female
	*n* (%)	*n* (%)	*n* (%)
20s	31 (100.0%)	6 (19.4%)	25 (80.6%)
30s	46 (100.0%)	19 (41.3%)	27 (58.7%)
40s	70 (100.0%)	31 (44.3%)	39 (55.7%)
50s	75 (100.0%)	33 (44.0%)	42 (56.0%)
60s	133 (100.0%)	63 (47.4%)	70 (52.6%)
70s	199 (100.0%)	95 (47.7%)	104 (52.3%)
> 80s	135 (100.0%)	87 (64.4%)	48 (35.6%)
Total	689 (100.0%)	334 (48.5%)	355 (51.5%)

### 2.3 Overview of the target area

Sera Town is located in a rural region, approximately an hour away from the central area by car. As of 2025, the total population of Sera Town is approximately 15,000 residents with 7,000 households (18). The gender distribution is 49.6% male and 52.4% female (18). The age distribution is as follows: 20s: 5.3%, 30s: 9.1%, 40s: 9.7%, 50s: 12.8%, 60s: 18.0%, 70s: 13.7%, 80s and above: 16.8% (18). The proportion of the individuals aged ≥65 years is 43.3% (18). Agriculture is the primary industry in the area (18).

### 2.4 Survey method

The survey was conducted using an anonymous self-administered questionnaire. Survey content was developed in collaboration with two university faculty members specializing in community nursing, as well as public health nurses. A pilot was conducted within members of the same laboratory, and the wording of questions and their answer choices were modified accordingly. The estimated completion time was approximately 5 min. The survey was conducted after the public health nurses responsible for its administration reviewed and confirmed the appropriateness of its content. The target population agreed to by returning the answered forms in an envelope. The survey was conducted from June 2024 to July 2024. The participants received a document explaining the purpose of the survey and how the survey results would be used and published. They confirmed their consent forms sent with questionnaires. Participants sending survey forms to the town hall meant to provide consent to participate in the study. This study was approved by the Ethics Committee of Hiroshima University (approval number: E-2024-0158).

### 2.5 Survey content

The survey included questions on the participants’ demographics, including age, gender, and family structure. To maintain confidentiality in the small community, age was reported in ranges. Questions also addressed participants’ health status, living conditions, and participation in community activities. Respondents rated their health status as “good,” “fairly good,” “average,” “not very good,” or “not good.” They rated their life satisfaction as “satisfied,” “somewhat satisfied,” “somewhat dissatisfied,” or “dissatisfied.” Japanese people tend to prefer ambiguous answers like “fairly” or “somewhat.” When they have a positive impression of a question, they often answer “average.” Isolation was measured using the Japanese version of the Lubben Social Network Scale-6 (LSNS-6) (19), with a score of <12 indicating isolation. Ten questions were made in accordance with the municipality’s wishes to understand the level of subjective feelings of loneliness (Supplementary Table 1).

#### 2.5.1 Participation in community activities

To examine participation in community activities, the types of community activities were categorized into Parent Teacher Association (PTA), neighborhood and residents’ associations, helping others, other volunteers, hobbies, and others. Participants selected activities in which they currently participated and those they would like to participate in the future. Participants chose “not applicable” if they “do not currently participate in any activity” or “do not want to participate in any activities.” We reported the number of individuals who participated in or expressed a desire to participate in each activity. The number of activities participated in and the number of activities desired were quantified for each survey respondent. Satisfaction with a community activity was answered by participants who participated in that activity or imagined participating in that activity. When participants viewed the activities they were involved in positively, they responded that they were “worthy.” Conversely, when they viewed the activities negatively, they responded “not worthy.” There were “non-respondents” who did not need to respond to the worthiness of the activity. All participants scored the worthiness of community activities as follows: 10 points for “worthy,” 5 points for “not worthy,” and 0 points for “not applicable.” The total score was divided by the number of activities to which the participants responded to calculate their average worthiness score (Supplementary Table 2).

#### 2.5.2 LSNS-6

The LSNS-6 was used as originally designed and consists of six questions. The LSNS was developed in 1988 to assess relationships among family, relatives, and friends (20). The scale is scored according to the number of family members, relatives, and friends with whom one can interact, discuss personal matters, or ask for help. The scale comprises six questions with six response options ranging from 0 (no one) to 5 (nine or more people). Total scores range from 0 to 30, with higher scores indicating greater social networks. A score of <12 indicates social isolation. The reliability and validity of the Japanese version of the LSNS-6 have been validated in Japanese population (Cronbach’s *α* = 0.82) (21). In our sample, the Cronbach’s α was 0.683. This score was referred to as the isolation score.

#### 2.5.3 Questionnaires on level of subjective feelings of loneliness

In this study, questionnaires on level of subjective feeling of loneliness were made in accordance with the municipality’s wishes to plan the town’s public health initiatives. It was made with the reference of previous studies (21, 22). The UCLA-LS, which was referenced, is a self-administered loneliness scale developed by Russell in 1978 (23). Its reliability and validity were confirmed in the third edition in 1996 (24). Cronbach’s *α* for the questionnaires we made was 0.894. Our made questionnaires’ total score ranged from 10 to 40. Higher total scores indicated greater level of subjective feelings of loneliness. This score was referred to as the subjective feelings of loneliness score.

### 2.6 Statistical analysis

#### 2.6.1 Identifying the characteristics of isolated people

Based on the isolation score, survey participants were categorized as “isolated” or “non-isolated.” Basic attributes, participation in community activities, and subjective feelings of loneliness scores were compared between the two groups. Participant characteristics, including age, gender, family structure, health status, and community activities, were evaluated using descriptive statistics. The χ^2^ tests, Kruskal–Wallis tests and or Mann–Whitney U test were used to confirm the association between these characteristics and isolation. Categorical items were divided for two-tailed analysis as follows: gender (male or female), age (≤59 or ≥60), household composition (living alone or not living alone), job (employed or unemployed), residential area (classified by median population), health status (not good; those who recognize “not good health status” or others; those who did not recognize “not good health status”), life satisfaction (satisfied or dissatisfied), medication status (yes or no), and frequency of going out (high or low). We grouped health status into “not good” and “others; not perceived as not good.” Japanese people tend to prefer vague responses, but they did not choose the term ‘average’ and rated their health poorly. This is significant. Life satisfaction was grouped as ‘satisfied’ for options containing the word ‘satisfied,’ and ‘dissatisfied’ for options containing the word ‘dissatisfied.’ Associations between these variables and isolation were examined using the χ^2^ tests and Kruskal–Wallis tests. Associations between subjective feelings of loneliness scores and isolation were assessed using the Mann–Whitney U test. Logistic regression analysis was then conducted using the survey items exhibiting significant differences as independent variables and isolation as the dependent variable. The independent variables included the following questions: “What kind of activity do you participate in?” “Do you feel worthiness in your activity?” “What kind of activities do you want to participate in in the future?” The dependent variable was represented as 1 for isolated and 0 for non-isolated. Statistical analyses were performed using SPSS Statistics for Windows version 25.0 (IBM Corp., Armonk, NY). Statistical significance was set at *p* < 0.05.

#### 2.6.2 Examining support measures for isolation

The status of community activity participation was assessed among isolated individuals. Isolated individuals were asked whether they currently participated in community activities, whether they would like to participate in the future, and whether they find these activities worthwhile. The relationship between the desire to participate in future community activities and the perceived worthiness of such activities was examined using the χ^2^ test.

## 3 Results

### 3.1 Participant characteristics

The characteristics of the study population are presented in Table 3. Most participants (67.8%) were in their 60s or older, lived with someone, and 73.4% were employed. Many lived in areas with large populations. Approximately 80% of the participants reported having a good health status and living a satisfactory life. More than half of the participants reported taking medications and experiencing illness. Most participants reported leaving the house for at least 1 day a week. The mean subjective feelings of loneliness score were 20.9 ± 5.9. 203 participants (105 males and 98 females) were classified as isolated, whereas 486 (229 males and 257 females) were not isolated. Not good health was reported by 12 (5.9%) and 8 (1.6%) of the isolated and non-isolated participants, respectively (*p* = 0.002). Dissatisfaction with life was reported by 18 (8.9%) and 9 (1.9%) of the isolated and non-isolated participants, respectively (*p* < 0.001). Infrequent outings from home were reported by 35 (17.2%) and 25 (5.1%) of the isolated and non-isolated participants, respectively (*p* < 0.001). The mean subjective feelings of loneliness scores were 25.25 ± 4.95 and 19.08 ± 5.24 for the isolated and non-isolated participants, respectively (*p* < 0.001).

**Table 3:** Characteristics of the study population.

Questions	Variables	Total (*N* = 689)	Isolated (*N* = 203)	Non isolated (*N* = 486)	χ^2^	*p*-value
Gender	Male	334 (48.5%)	105 (51.7%)	229 (47.1%)	1.216	0.270^#^
	Female	355 (51.5%)	98 (48.3%)	257 (52.9%)		
Age	≤59 years	222 (32.2%)	72 (35.5%)	150 (30.9%)	1.390	0.238^#^
	≥60 years	467 (67.8%)	131 (64.5%)	336 (69.1%)		
Family structure	One person living alone	82 (11.9%)	26 (12.8%)	56 (11.5%)	0.226	0.635^#^
	Multiple people	607(88.1%)	177 (87.2%)	430 (88.5%)		
Job status	Employed	506 (73.4%)	143 (70.4%)	363 (74.7%)	1.325	0.250^#^
	Unemployed	183 (26.6%)	60 (29.6%)	123 (25.3%)		
Living area	Populated	520 (75.5%)	150 (73.9%)	370 (76.1%)	0.388	0.533^#^
	Less populated	169 (24.5%)	53 (26.1%)	116 (23.9%)		
Health status	Good	117 (17.0%)	30 (14.8%)	87 (17.9%)	16.783	0.002^#^
	Fairly good	155 (22.5%)	35 (17.2%)	120 (24.7%)		
	Average	276 (40.0%)	81 (39.9%)	195 (40.2%)		
	Not very good	121 (17.6%)	45 (22.2%)	76 (15.6%)		
	Not good	20 (2.9%)	12 (5.9%)	8 (1.6%)		
Life satisfaction	Satisfied	183 (26.6%)	27 (13.3%)	156 (32.1%)	46.136	< 0.001^#^
	Somewhat satisfied	363 (52.7%)	109 (53.7%)	254 (52.2%)		
	Somewhat dissatisfied	116 (16.8%)	49 (24.1%)	67 (13.8%)		
	Dissatisfied	27 (3.9%)	18 (8.9%)	9 (1.9%)		
Do you take medicine?	Yes	432 (62.7%)	126 (62.1%)	306 (63.0%)	0.049	0.825^#^
	No	257 (37.3%)	77 (37.9%)	180 (37.0%)		
Past medical history	Yes	394 (57.2%)	115 (56.7%)	279 (57.4%)	0.034	0.855^#^
	No	295 (42.8%)	88 (43.3%)	207 (42.6%)		
Frequency of going out	At least once a week	629 (91.3%)	168 (82.8%)	461 (94.9%)	26.359	<0.001^#^
	Less than once a week	60 (8.7%)	35 (17.2%)	25 (5.1%)		
Loneliness Score		20.9 ± 5.9	25.25 ± 4.95	19.08 ± 5.24		<0.001^&^
What kind of activity do you participate in?	At least one	396 (57.5%)	74 (36.5%)	322 (66.3%)	52.033	<0.001^#^
What kind of activities do you want to participate in?	At least one	326 (47.3%)	83 (40.9%)	243 (50.0%)	51.237	0.029^#^
Do you feel worthiness in your activity?	At least one	369 (53.6%)	66 (32.5%)	303 (62.3%)	4.771	<0.001^#^

#, Chi–squared test and Kruskal–Wallis test; &, Mann–Whitney U test.

Participation in community activities was reported by 36.5 and 66.3% of the isolated and non-isolated participants, respectively (*p* < 0.001). Significant differences in participation in all activities were observed between the isolated and non-isolated participants. Furthermore, 32.5% of the isolated and 62.3% of the non-isolated participants considered these activities worthwhile (p < 0.001). Significant differences were observed for satisfaction with all activities between the isolated and non-isolated participants. Notably, 40.9% of the isolated and 50% of the non-isolated participants desired to participate in future community activities (*p* = 0.029).

### 3.2 Comprehensive associations of isolation and states

**Figure 1:**
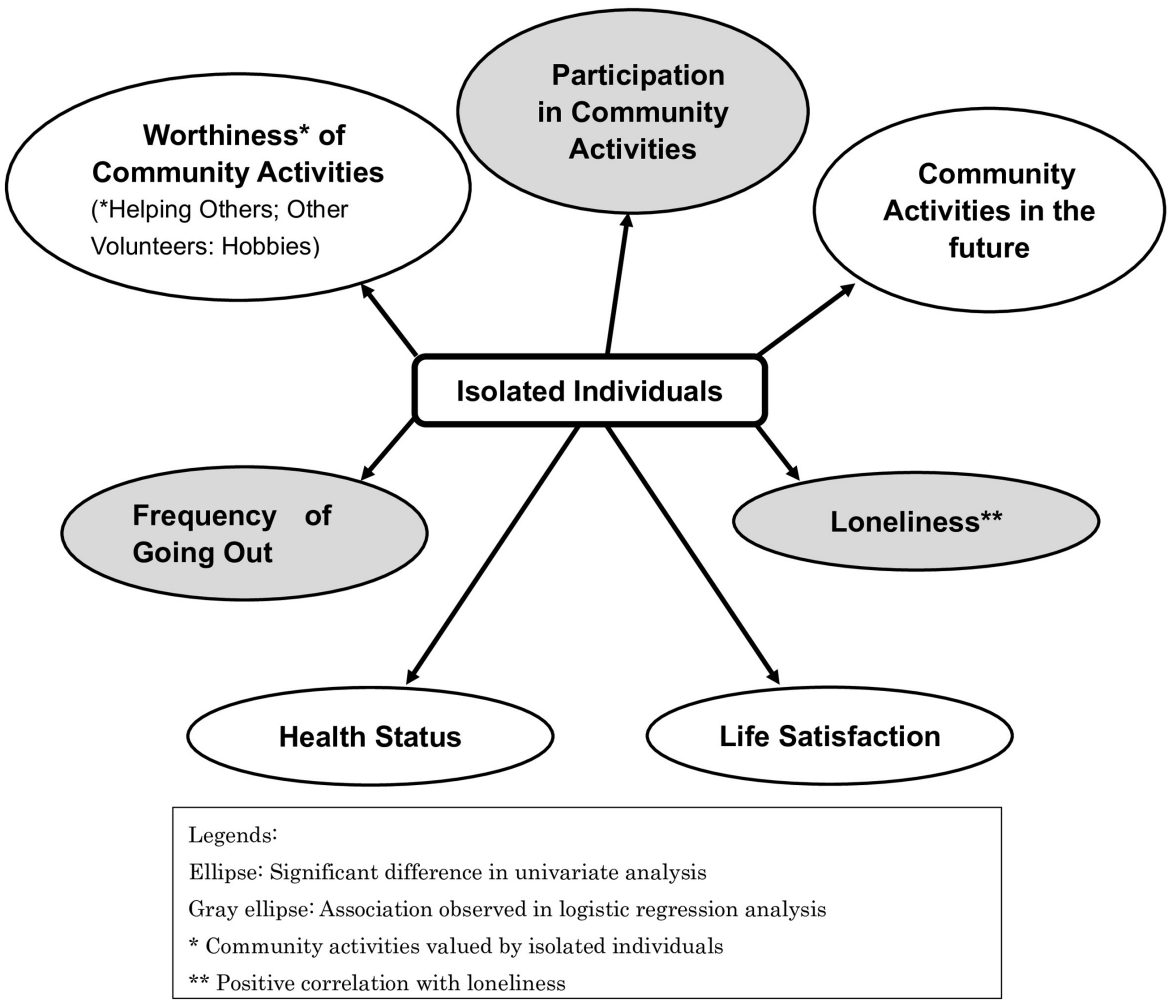
States associated with isolated individual.

The variable subtraction method with a likelihood ratio test was used to select the variables. Gender and age were forced input as demographic data. States related to isolation included the frequency of going out (odds ratio [OR] = 0.434, 95% CI [confidence interval]: 0.227–0.832, *p* = 0.012), subjective feelings of loneliness score (OR = 1.223, 95% CI: 1.169–1.278, *p* < 0.001), and participation in activities (OR = 0.531, 95% CI: 0.361–0.781, *p* = 0.001). The Hosmer–Lemeshow test results for this model were compatible at *p* = 0.854, with a discriminant predictive value of 76.8% for the predicted and measured values. The results of logistic regression models are presented in Table 4. Figure 1 shows the states associated with isolated individuals. Ellipses indicate items showing significant differences in the univariate analysis. Gray ellipses indicate variables for which an association was observed in the logistic regression analysis. * marks community activities valued by isolated individuals, and ** marks a positive correlation between isolation and subjective feelings of loneliness.

**Table 4:** Logistic regression analysis for identifying comprehensive relationships between isolation and states.

Variable	Category	B	OR	95% CI		p-value
				Lower	Upper	
Gender	Male (1)/Female (0)	0.065	1.067	0.716	1.589	0.750
Age	≥60 years (1)/ ≤59 years (0)	−0.151	0.860	0.555	1.333	0.499
Health Status	Not good (1)/Others (0)	−0.018	0.982	0.595	1.622	0.944
Life satisfaction	Dissatisfied (1)/Satisfied (0)	0.149	1.160	0.723	1.863	0.538
Frequency of going out	At least once a week (1)/Less than once a week (0)	−0.834	0.434	0.227	0.832	0.012
Loneliness score		0.201	1.223	1.169	1.278	<0.001
What kind of activity do you participate in?		−0.633	0.531	0.361	0.781	0.001
Do you feel worthiness in your activity?		0.035	1.036	0.968	1.108	0.305
What kind of activities do you want to participate in in the future?		0.201	0.906	0.690	1.190	0.476

Hosmer–Lemeshow test; χ^2^ = 4.034; *p* = 0.854; Nagelkerke R^2^ = 0.369; Predictive accuracy = 76.8%, *n* = 689; OR, odds ratio; CI, confidence interval.

### 3.3 Community activity participation and perceived value among isolated participants

Table 5 presents the characteristics of community activities engaging isolated participants. Notably, 63.5% of individuals did not participate in any activities. Among the isolated participants, 24.1% participated in activities such as PTA, neighborhood, and residents’ associations, and 12.8% engaged in hobbies. Furthermore, 59.1% expressed no desire to participate in any activity, whereas 30.5% wanted to participate in hobbies. Participants who answered “yes” to the question, “Do you feel worthiness in your activity?” were involved in the following types of activities: 33 (16.3%) in PTA, neighborhoods, and residents’ associations; 15 (7.4%) in helping others; 13 (6.4%) in other volunteers; 37 (18.2%) in hobbies; and 7 (3.4%) in others. Table 6 presents the relationship between worthiness and future community activities. Participants who felt worthiness in helping others, other volunteers, and hobbies desired to participate in these activities significantly (*p* = 0.025, *p* = 0.040, and *p* = 0.007, respectively).

**Table 5:** Characteristics of community activities for isolated people (*N* = 203).

Questions	Activities	Yes *n* (%)
What kind of activity do you participate in? (multiple answers)	PTA, neighborhood, and residents’ associations	49 (24.1%)
	Helping others	8 (3.9%)
	Other volunteers	4 (2.0%)
	Hobbies	26 (12.8%)
	Others	5 (2.5%)
	Nothing	129 (63.5%)
What kind of activities do you want to participate in in the future? (multiple answers)	PTA, neighborhood, and residents’ associations	14 (6.9%)
	Helping others	12 (5.9%)
	Other volunteers	13 (6.4%)
	Hobbies	62 (30.5%)
	Others	4 (2.0%)
	Nothing	120 (59.1%)
Do you feel worthiness in your activity? (multiple answers)	PTA, neighborhood, and residents’ associations	33 (16.3%)
	Helping others	15 (7.4%)
	Other volunteers	13 (6.4%)
	Hobbies	37 (18.2%)
	Others	7 (3.4%)

**Table 6:** Relationship between worthiness and future community activities.

Activities	Do you feel worthiness in your activity?	Do you want to participate in the future?		χ^2^	*p*-value
		Yes *n* (%)	No *n* (%)		
PTA, neighborhood, and residents’ associations (*N* = 73)	Yes	3 (42.9%)	30 (45.5%)	0.017	0.896
	No	4 (57.1%)	36 (54.5%)		
Helping others (*N* = 38)	Yes	3 (100.0%)	12 (34.3%)	4.994	0.025
	No	0 (0.0%)	23 (65.7%)		
Other volunteers (*N* = 39)	Yes	2 (100.0%)	11 (29.7%)	4.216	0.040
	No	0 (0.0%)	26 (70.3%)		
Hobbies (*N* = 61)	Yes	17 (85.0%)	20 (48.8%)	7.390	0.007
	No	3 (15.0%)	21 (51.2%)		
Others (*N* = 43)	Yes	0 (0.0%)	7 (16.7%)	0.199	0.655
	No	1 (100.0%)	35 (83.3%)		

## 4 Discussion

### 4.1 Main findings

This study identified the characteristics of the isolated individuals. The analysis revealed significant associations between isolation and health status, life satisfaction, frequency of going out, level of subjective feelings of loneliness, and participation in community activities. Furthermore, isolation suggested to be to associated with outings, levels of subjective feeling of loneliness, and participation in community activities. These findings suggest that isolation is associated with less frequent outings, higher levels of subjective feelings of loneliness, and lower participation in community activities. Our results also reveal that isolation is associated not only with the psychology of the participant but also with participation in community activities. A detailed consideration of community activities could lead to propose activities that are easy for isolated individuals to participate in.

### 4.2 Support for isolation considering rural characteristics

The study area was a rural region distant from the city center, with a high proportion of older adults. The survey response rate was high among females in their 70s, males in their 70s, and males in their 80s. Conversely, the response rate was low among younger males and super-older females, making their isolation status unclear and making it difficult to say the findings accurately reflect their characteristics. The percentage of respondents who returned the mailed survey was higher among females than males and higher among older adults than younger people. This result means that the focus is on the isolation of older adults which is characteristics of the target area. Continuous health dialogues in rural areas effectively reduce subjective feelings of loneliness and improve health outcomes by promoting regular interactions and building supportive networks (25). When older adults attempt to go out, physical decline, the inability to secure support, and distance being too far may cause them to abandon their plans. Digital-based isolation support can provide opportunities for communication with others. A previous study found that direct interaction with peers using digital means is effective in improving subjective feelings of loneliness in the older adults (26); however, the continually developing digital technology is considered difficult for the current middle-aged and older populations. Using digital technology to send administrative communications at a level that encourages participants to join activities, combined with face-to-face interactions, has been suggested to be effective in countering isolation in rural areas (27). These findings are consistent with evidence showing that s and related psychological factors significantly affect well-being and quality of life among older adults (28).

### 4.3 Relationships between isolation and social characteristics

Isolated individuals significantly reported feeling lonely. The relationship between isolation and subjective feelings of loneliness has been reported in previous studies (13, 29) and was also observed in this study. Interventions for isolated individuals may influence subjective feelings of loneliness. Supporters should actively guide isolated individuals to solve their health concerns. As the emotions of isolated individuals are likely to fluctuate with age and health status, supporters need to remain engaged with them and notice changes. It is important for public health to solve the health problems of isolated individuals. Isolated individuals were more likely to have poor health. They may have been unable to go out due to their health status, or they may have deliberately avoided seeking medical care to avoid social interaction. Isolated individuals may have few opportunities to review their health status. To manage the health of isolated individuals, cooperation between administrations and hospitals is essential. A lack of participation in community activities is associated with a lack of opportunities for health checks (30). However isolated individuals had significantly low participation in community activities, identifying individuals based on their history of hospital visits will be able to get an opportunity to review their own health with others with shared experiences. Community activities may provide isolated individuals with opportunities to reassess their health status. Isolated individuals were more dissatisfied with their lives than non-isolated individuals. A previous study reported greater life satisfaction among individuals with larger social networks (31). Having a large social network can facilitate daily consultations and access to support during emergencies. Building trusting relationships with neighbors enhances a sense of safety and reduces the feeling of dissatisfaction. Isolated individuals tend to have limited communication with friends and may not feel safe or satisfied in daily life. Therefore, supporters must identify isolated individuals based on objective indicators and proactively discuss their life problems with them.

In this study, isolated individuals reported going out significantly less frequently than non-isolated individuals. Furthermore, the frequency of going out was associated with isolation comprehensively. We assessed isolation using an isolation score based on the number of family, relatives, and friends the individual interacted with regularly. A lower frequency of outings among isolated individuals indicated fewer opportunities for social interaction, they rarely communicated with others and were more likely to remain alone. Reduced frequency of outings has also been associated with the decreased frequency of daily conversations, which may increase the risk of dementia and depression (32). Consequently, isolated individuals may exhibit a higher risk of mental problems because of limited daily interaction. Therefore, promoting communication is critical to mitigating health risks. For example, staff working in community facilities that are regularly used by people should maintain routine engagement with visitors. This behavior helps identify an emergency when staff notice that the individual is no longer present. Additionally, isolated individuals often require more time to build trusting relationships with people around them. Community activity leaders, who are proactive and have many friends, may share common ground with participants, such as living in the same neighborhood, making it easier to build trusting relationships.

The participation of isolated individuals in community activities was observed to be significantly poor in the present study. Participation in community activities and isolation are suggested to exhibit a comprehensive relationship. Community activities must have interesting features to attract those who have not yet attended them. The content of the activities, ability to participate with friends, and interaction with participants encourage continued participation in activities (33). Invitations by others help isolated individuals participate in community activities. In this study isolated individuals often participated in PTA, neighborhood, and residents’ associations and hobbies. Connecting new activities to existing or familiar activities in which the isolated individuals have already participated could further improve their participation.

### 4.4 Community activities for isolated individuals

Previous studies reported that isolated individuals are less likely to participate in activities and engage in interesting activities (33). Worthiness was associated with future community activities such as helping others, other volunteers, and hobbies. Helping others and other volunteers are activities in which individuals feel their efforts are useful to others. Also hobbies bring pleasure to life. Isolated individuals may prefer community activities that clarify their purpose and involve shared pleasure with others. Furthermore, participation in social activities may also increase healthy life expectancy (34). This study suggests that isolated individuals may prefer community activities that clarify their purpose and involve shared pleasure with others. Municipalities must identify local resources, community leaders, and mutual support among residents in a Community-based Integrated Care System. Municipalities and community health workers play a role in facilitating and supporting residents as they proactively promote community activities. When considering the activities that residents should undertake, they can jointly think about and share the purpose of the activities. Furthermore, they can identify local resources and recommend them to those in need. Isolated individuals are also human resources within the community, and nurturing them is a public welfare responsibility of the community.

### 4.5 Study limitations

This study has some limitations. First, the study was conducted in a small, rural and single town, and write that this limits the generalizability of the findings. Participants were limited to those who completed the survey forms and returned the survey forms. In this study, young males and super-older females did not respond to the survey, so their status of isolation could not be determined. Further investigations are required to clarify these characteristics. The high proportion of older adults, which is a characteristic of rural areas, was reflected in the characteristics of isolation. Our findings suggest that isolation solutions can be applied to other rural areas. Second, questionnaires on subjective feelings of loneliness were made to gain reasons to programs that facilitate participation in health activities when the municipality plans the town’s public health initiatives. The feelings of loneliness experienced by the participants may have been measured at low levels. In additionally, the study is based on participants’ self-report, which may lead to recall and social desirability bias. Nevertheless, this study identified the potential solution to addressing isolation by focusing on community activities tailored to the characteristics of isolated individuals. Proposing community activities tailored to the characteristics of isolated individuals helps those experiencing low levels of subjective feeling of loneliness maybe only less severe forms of subjective feeling of loneliness participate more easily. Future studies should involve the larger datasets and categorize the types of community activities to enhance the analysis. The effectiveness of community activities should be examined by measuring the changes in each individual who participates in the long term.

## 5 Conclusion

Isolated individuals were characterized by poor health, infrequent outings, and a lack of participation in community activities. They also reported feeling dissatisfied with their lives and experiencing subjective feelings of loneliness. The frequency of going out, subjective feelings of loneliness, and participation in community activities were suggested to be comprehensively associated with isolation. Community activities in which isolated individuals want to participate were suggested to have clear goals such as helping others and hobbies.

## Data Availability

The datasets presented in this article are not readily available because of ethical restrictions. Requests to access the datasets should be directed to HK, khiromi@hiroshima-u.ac.jp.
