# Supplementary material for: Community activities and their association with social isolation in rural Japan
Source: Front Public Health. 2026 Jan 5;13:1697377. doi: 10.3389/fpubh.2025.1697377 (PMC12812739; doi:10.3389/fpubh.2025.1697377)
Supplement: Supplementary file 1 [file Table_1.DOCX]

Supplementary Table1. Questionnaires on level of subjective feelings of loneliness

| Questions | Answers |
| --- | --- |
| Do you feel that you fit in with the people around you? | - I always feel that. - I sometimes feel that. - I don't feel that so much. - I don't feel that at all. |
| Do you ever feel like you do not have relationships with others? |  |
| Do you ever feel that you have a lot in common with the people around you? |  |
| Do you feel that you are sociable and easy to get along with? |  |
| Do you feel that you have close friends? |  |
| Have you ever felt that interacting with others is meaningless? |  |
| Do you feel that you could find like-minded friends if you wanted to? |  |
| Do you feel that there are people who truly understand you? |  |
| Do you feel that you have someone to talk to? |  |
| Do you feel that you have someone you can rely on? |  |
