# Supplementary material for: Community activities and their association with social isolation in rural Japan
Source: Front Public Health. 2026 Jan 5;13:1697377. doi: 10.3389/fpubh.2025.1697377 (PMC12812739; doi:10.3389/fpubh.2025.1697377)
Supplement: Supplementary file 2 [file Table_2.docx]

Supplementary Table 2. How to calculate the worthiness score of community activities?

| Community Activities | Worthiness | Score* | Number of activities responded** |
| --- | --- | --- | --- |
| - PTA, neighborhood and residents’ associations - Helping others - Other volunteers - Hobbies - Others; class reunions, religious activities, etc. | I find that activity very worthy of me. | 10 | /0~5 |
|  | I find that activity worthy of me. |  |  |
|  | I don’t find that activity very worthy of me. | 5 |  |
|  | I don’t find that activity worthy of me. |  |  |
|  | I don’t participate in the activity. | 0 |  |

*: Add the points for each activity.

**: Divide the total score by the number of activities responded.
